# Thermal Model for Timber Fire Exposure with Moving Boundary

**DOI:** 10.3390/ma14030574

**Published:** 2021-01-26

**Authors:** Stanislav Šulc, Vít Šmilauer, František Wald

**Affiliations:** 1 Faculty of Civil Engineering, Department of Mechanics, Czech Technical University in Prague, Thákurova 7, 166 29 Prague, Czech Republic; vit.smilauer@fsv.cvut.cz; 2 Faculty of Civil Engineering, Department of Steel and Timber Structures, Czech Technical University in Prague, Thákurova 7, 166 29 Prague, Czech Republic; wald@fsv.cvut.cz

**Keywords:** charring rate, timber, advancing front, moving boundary condition, burnout, adiabatic surface temperature, model

## Abstract

Fire exposure of timber leads to charring, surface cracking and timber burnout, shifting the external thermal load deeper into the timber domain. This phenomenon plays its role mainly in situations of longer fire exposure. The majority of current approaches and models assume initial geometry during the whole analysis, leading generally to the overestimation of the insulation effect of the charred layer and to a limited burnout. This paper presents a heat transport model which is supplemented with a moving boundary condition, a criterion for the finite element deactivation and the internal heat source. Comparison with experiments using a constant radiative load testifies that the moving boundary condition becomes important after approximately 10 min of fire exposure and rather leads to a constant charring rate observed in several experiments.

## 1. Introduction

Timber exposure to high temperatures triggers several multiscale thermochemical processes responsible for the timber degradation and loss of mechanical resistance [1]. The charring rate, β, is a traditional way of expressing degradation experimentally from two types of test setups. The first one uses time–temperature loading curves such as ISO 834 or ASTM E 119, while the second one takes advantage of a constant radiation source like a gas-fired radiant panel [2].

The classical approach for assessing the charring rate relies on thermal analysis, which assumes that timber turns to char on reaching approximately 300 °C, coinciding with the onset of mechanical properties degradation [3,4,5]. The charring rate provides explicit information about the temperature propagation during the fire exposure.

Thermal and coupled models can tackle the multiphysical nature of charring, including the temperature, humidity or internal heat source [3,6]. The charring rate becomes a traditional validation parameter, taking into account the boundary conditions, timber properties and other factors [7,8,9]. The most advanced models introduce heterogeneous kinetics for both pyrolysis (thin interface between char and wood with no oxidation) and oxidation (converting cellulose, hemi-cellulose and lignin to char and finally to ash) [1,10,11]. Those models can provide an accurate description of ongoing processes, however, they need to solve conservation equations for mass, species, energy and momentum, giving rise to a considerable amount of parameters and necessary calibrations.

The definition of correct boundary conditions is crucial for validation. A constant heat flux on the timber surface is difficult to achieve even from a constant distant source since the temperature of the exposed surface rapidly increases and the flux drops. In a simple form, the heat flux across an exposed surface can be approximated as

(1) $$q=\varepsilon \sigma \left(\sum \varepsilon_{i}F_{i}T_{\mathrm{inc},i}^{4}-T_{S}^{4}\right)+h\left(T_{g}-T_{S}\right)$$

where $T_{S}$ is the temperature of the exposed timber surface, ε is its emissivity, *h* is the heat transfer coefficient, σ is the Stefan-Boltzmann constant and $T_{g}$ is the gas temperature. Different radiative sources, $T_{\mathrm{inc},i}$, contribute to the flow with its emissivity $\varepsilon_{i}$ and the view factor $F_{i}$. Equation (1) can be expressed in a simpler form using adiabatic surface temperature (AST) concept [12,13,14,15]

(2) $$q=\underset{radiation}{\underset{︸}{\varepsilon \sigma \left(T_{\mathrm{AST}}^{4}-T_{S}^{4}\right)}}+\underset{convection}{\underset{︸}{h\left(T_{\mathrm{AST}}-T_{S}\right)}}$$

where $T_{\mathrm{AST}}$ is the value governing both radiative and convective boundary conditions. The constant $T_{\mathrm{AST}}$ is a suitable representation of the thermal effect of a radiative panel with a constant power [15] and can be further used for the coupling between CFD and the thermal model [13,16].

**Figure 1:**
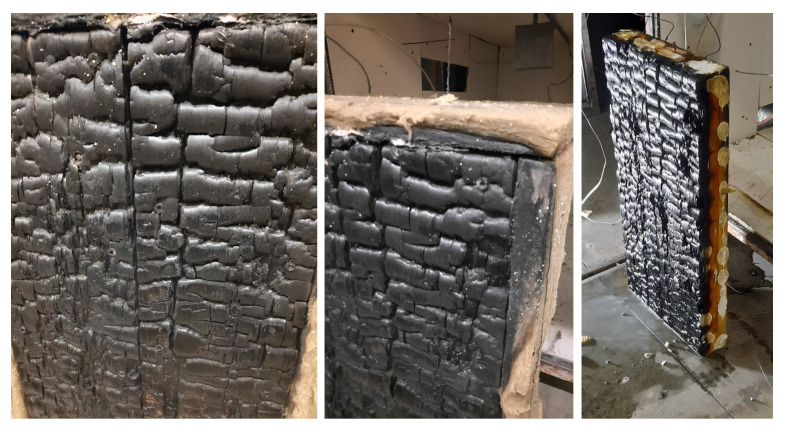
Timber panel after 30 min of ISO 834 exposure.

The majority of current models neglect further degradation of a charred layer, occurring especially on longer exposures and resulting to a moving boundary. Cracking, embrittlement and falling off leads to effective thinning of the charred layer, decreasing its thermal insulation. To illustrate that fact, Figure 1 presents the state of a timber panel after 30 min of ISO 834 fire exposure. It displays wide cracks and regions where the charred layer has partially disappeared.

**Figure 2:**
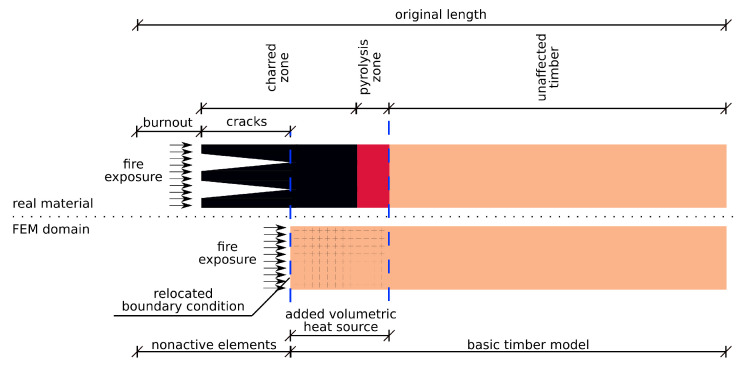
Fire exposure of timber, charred zone and a moving boundary condition.

During the charring process, the charring front penetrates deeper from the exposed surface as sketched in Figure 2. The energy stored in the timber becomes released and it causes a further temperature increase with a positive feedback. The char layer often cracks due to temperature-induced restrained strain with buckling [17], a further crack increase originates from the mass loss [18,19]. Cracks 3–6 mm wide were reported on a 38 mm deep char layer [19].

The main objective of this paper aims at capturing a thinning charred layer using a moving boundary condition (MBC) and an internal heat source in the framework of finite element analysis for heat transport. Further validations clearly show a pronounced effect of the effective charred layer on accelerating heat ingress.

A temperature-based criterion specifies conditions for removing excessively charred and cracked regions, see Figure 2. A virtual moving boundary captures the effect of burnout and cracking to an effective depth and allows modeling more accurately longer exposures when a superficial charred zone cracks or disappears.

The internal heat source, mimicking internal burning, is another model’s extension. The source captures better situations of different external radiative loads; experiments showed a monotonous relationship between the power of the external radiative load and the charring rate [7,20,21]. Further, experiments by Kashiwagi et al. [22] showed that the oxygen concentration increases the charring rate, related to internal heat release.

## 2. Thermal Model with Moving Boundary Condition

The thermal model is based on a heat conduction equation for isotropic material

(3) $$\begin{array}{ccc} -\nabla^{T}q(x,T)+Q_{\mathrm{vol}}\left(x,T\right) & = & \rho \left(x,T\right)c_{V}\left(x,T\right)\frac{\partial T(x,t)}{\partial t} \end{array}$$

(4) $$\begin{array}{ccc} q(x,T) & = & -\lambda (x,T)\nabla T(x) \end{array}$$

where λ(x) [Wm${}^{-1}$K${}^{-1}$] is the thermal conductivity of an isotropic material, ρ(x) is the bulk density, $c_{V}\left(x\right)$ is the thermal capacity and $Q_{\mathrm{vol}}$ is the volumetric internal heat source $Q_{\mathrm{vol}}$ [W·m${}^{-3}$]. Equation (3) describes the behavior inside the domain. Convection and radiation boundary conditions are applied on its boundaries from Equation (2), both governed by the $T_{\mathrm{AST}}$.

The current implementation activates $Q_{\mathrm{vol}}$ once the temperature exceeds 300 °C, coinciding with the pyrolysis initiation [3,5]. The upper estimate of $Q_{\mathrm{vol}}$ stems from the combustion heat, which is approximately 20 MJ/kg for dry wood [23]. Approximately two thirds of the heat of combustion is released by flaming, which we consider as the energy leaving the timber domain, while approximately one third is released by smouldering [24], which could be partly considered as the internal heat source. The simulations in Section 3 show that MBC maintained $Q_{\mathrm{vol}}$ active under 15 min. In this particular case, the theoretically admissible value is $\frac{1}{3}$20 × 10${}^{3}$/(15 × 60) = 7.41 kW/kg, which leads to $Q_{\mathrm{vol}}^{\max}$ = 3703 kW/m${}^{3}$ for timber’s dry bulk density of 500 kg/m${}^{3}$. Reasonable results were obtained in further models with $Q_{\mathrm{vol}}$ up to 500 kW/m${}^{3}$, which is 13.5% of the theoretical smouldering value.

**Figure 3:**
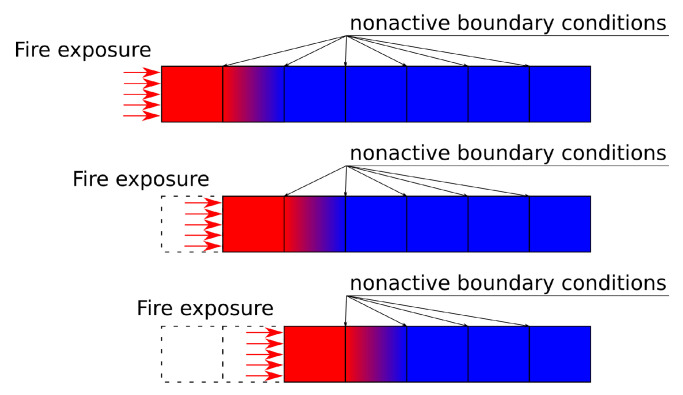
Implementation of a moving boundary condition.

The moving boundary condition is implemented via the deactivation of superficial finite elements, see Figure 3. This causes the removal of a finite element and the activation of a newly exposed surface with the boundary condition. The criterion for deactivation is described below.

### 2.1. Criterion for Finite Element Deactivation

The criterion should be based on the limit temperature, $T_{lim}$, acting on a certain volume of a finite element. Otherwise, the results become highly mesh-dependent. Generally, the temperature on a finite element is approximated as

(5) $$\begin{array}{c} T(x)=N(x)\cdot r \end{array}$$

with the interpolation matrix N(x). A mean temperature over an element could be conveniently evaluated using the Gauss integration points on curvilinear (isoparametric) elements, considering a corresponding polynomial interpolation degree and integration schema. This can be written as

(6) $$\begin{array}{c} T_{mean}=\frac{1}{V}\int_{V}T\left(x\right)dV=\frac{1}{V}\sum_{i=0}^{i=M}w_{i}T\left(s_{i}\right)\left|J_{i}\right|=\frac{1}{V}\left(\underset{N\;\mathrm{highest}\;\mathrm{temperatures}}{\underset{︸}{\sum_{i=0}^{i=N}w_{i}T_{i}\left(s_{i}\right)\left|J_{i}\right|}}+\sum_{i=N+1}^{i=M}w_{i}T_{i}\left(s_{i}\right)\left|J_{i}\right|\right) \end{array}$$

where the term “*N* highest temperatures” identifies the integration points where the temperature exceeds $T_{lim}$. Since $\sum_{i=0}^{M}w_{i}=1$, a partial sum of several weights corresponds to a spatial fraction over a finite element. The criterion uses existing integration points on a finite element and a user needs to specify $T_{lim}$ and the critical spatial fraction P${}_{cr}$, so the element deactivation happens when

(7) $$\sum_{i=0}^{i=M}\left[w_{i}\;\;\mathrm{if}\;\;\;\left(T_{i}\geq T_{lim}\right)\right]\geq P_{cr}$$

A quadrilateral element with four integration points may serve as a classic example, assigning the weights of 0.25 to each integration point. Setting N=1 requires that $T_{lim}$ is reached in one point, corresponding exactly to a quarter area in rectangular elements. N=2 requires $T_{lim}$ in two points etc. Another example covers a hexahedral element with a quadratic temperature approximation and a 3×3×3 array of integration points used in the simulations below. Setting N=1 for a corner point yields $w_{1}={\left(5/18\right)}^{3}=0.0214$, N=2 results in $w_{2}=w_{1}+{\left(5/18\right)}^{2}\cdot 4/9=0.0556$. Calibrations in the paper show that $P_{cr}=0.32$ yields a reasonable response with regards to the element size and the mesh objectivity. If $P_{cr}<0.32$, it causes an inappropriate acceleration of MBC from the corner of a 2D domain.

### 2.2. Material Properties

**Figure 4:**
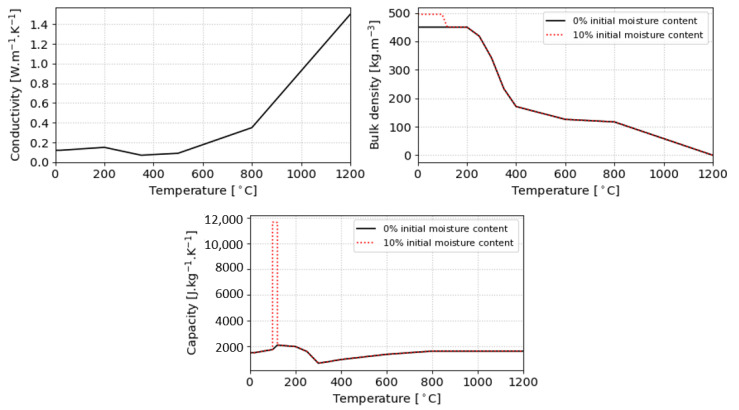
Timber temperature-dependent properties according to EC5 for timber dry bulk density 450 kg/m${}^{3}$ and initial moisture contents 0% and 10%.

The solution of Equation (3) requires material thermal properties, preferably temperature dependent. For simplicity reasons, we adopt the definition from EN 1995-1-2:2006 [25] (EC5), particularly for heat conductivity, thermal capacity and bulk density, see Figure 4. The thermal conductivity is the same for any initial moisture content. Exposures exceeding 30 min are advised to be used with caution according to the EC5 validity ranges.

### 2.3. Radiative Source

**Figure 5:**
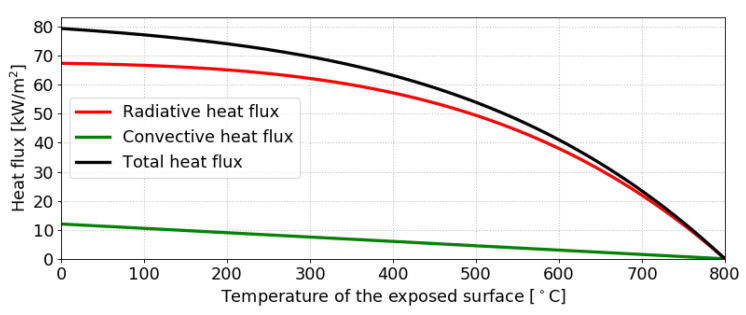
Heat flux across an exposed surface for $T_{\mathrm{AST}}$ = 800 °C, ε = 0.9 and h = 15 W/m${}^{2}$/K.

As mentioned in the introduction, a constant $T_{\mathrm{AST}}$ can capture the effect of a constant radiative source. To exemplify the situation, Figure 5 shows the heat fluxes for $T_{\mathrm{AST}}$ = 800 °C. In such a common case, the radiative flux plays a dominant role during arbitrary $T_{\mathrm{AST}}$.

### 2.4. Impact of Internal Heat and Moving Boundary

A simple 1D task can demonstrate easily the impact of the internal heat source (IHS) and moving boundary conditions (MBC) applied separately. A semi-infinite domain is exposed to $T_{\mathrm{AST}}$ = 650 °C on the left surface The emissivity of the surface was considered as 0.9 and the heat transfer coefficient as 20 Wm${}^{-1}$K${}^{-1}$. The initial temperature of the domain was set to 0 °C, $T_{lim}$ = 630 °C, $Q_{\mathrm{vol}}$ = 500 kW/m${}^{3}$. A finite element had the dimensions of 1×1×1 mm.

**Figure 6:**
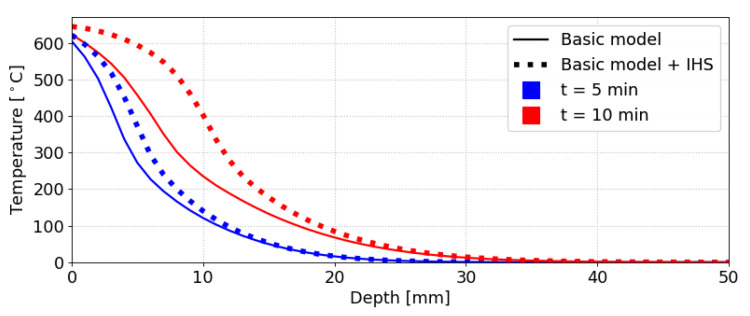
Example of the effect of the internal heat source (IHS). Semi-infinite domain.

Figure 6 shows the impact of IHS alone. It increases the temperature profile and indirectly accelerates charring, apparent after 10 min. IHS itself would still not suffice to capture the charring rate acceleration on increasing the radiative load.

**Figure 7:**
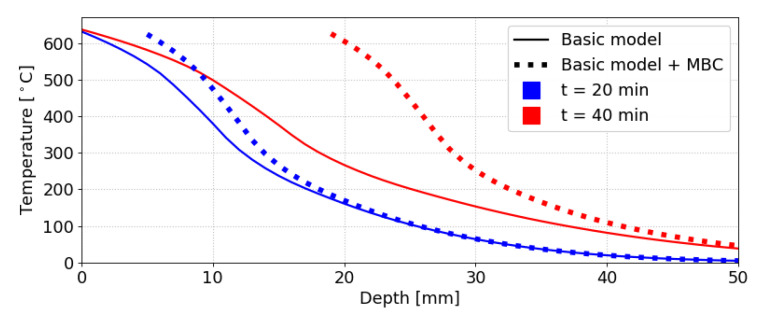
Example of the effect of a moving boundary condition (MBC). Semi-infinite domain.

The moving boundary condition causes a stronger increase in temperature with regards to the initial unchanged configuration. The effect is shown in Figure 7. MBC rather leads to a constant charring rate due to the advancing front, corresponding better to experimental results; Section 3 further demonstrates this effect.

## 3. Results and Discussion

The results are based on solving Equations (3) and (4). Two unknown parameters $T_{lim}$, $Q_{\mathrm{vol}}$ are calibrated from experimental data.

All simulations considered $T_{\mathrm{AST}}$ as a boundary condition, determined from the radiative heat flux component only and neglecting the convective term. During 1D simulations, the heat flow into timber also considered only the radiative term from Equation (2).

### 3.1. Experiment by Kashiwagi et al.

**Figure 8:**
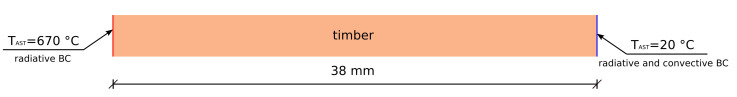
Geometry of Kashiwagi’s et al. experiment.

Kashiwagi et al. [22] performed a 1D experiment on a timber element exposed to a radiative load from one side, see the Figure 8.

A radiative power of 40 kW/m${}^{2}$ was applied through a water-cooled pipe and a window to eliminate convection. Equation (2) without the convective part yields $T_{\mathrm{AST}}$ = 670 °C with ε = 0.9. The heat transfer coefficient of the non-exposed surface was assumed as 15 W/m${}^{2}$/K. The initial temperature was 20 °C, the timber dry bulk density 380 kg/m${}^{3}$, the initial moisture content 5 wt. % and the material properties followed Figure 4. The model settings were $T_{lim}$ = 640 °C and $Q_{\mathrm{vol}}$ = 100 kW/m${}^{3}$.

**Figure 9:**
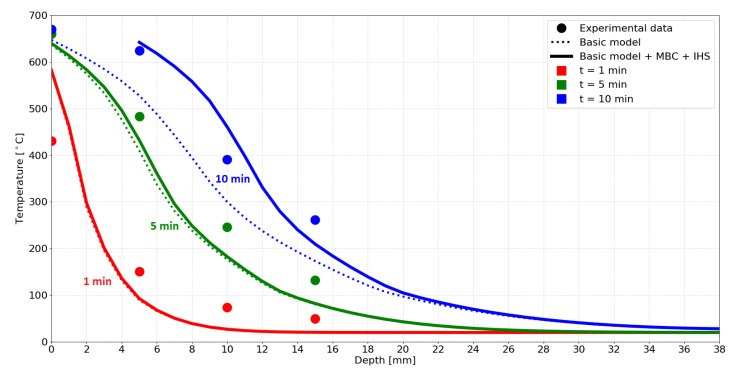
Simulation results compared with experimental data by Kashiwagi et al. [22].

The results depicted in Figure 9 present a good match with the experimental data. The values measured at the surface confirm the value of $T_{\mathrm{AST}}$, with an exception at 1 min, caused by the missing convective boundary condition. In the experiment, the ambient air lowers the surface temperature until the timber starts charring.

The effect of MBC becomes apparent at 10 min, showing that it is a necessary component of the model to achieve the experimental temperature distribution. Slightly higher temperatures inside the timber are most likely caused by the assigned material properties from Figure 4.

### 3.2. Experiment by Reszka and Torero

**Figure 10:**
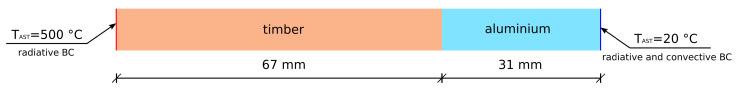
Experiment setup according to [26].

Reszka and Torero [26] tested a timber member attached to an aluminium base, see Figure 10.

The thermal load was described as a heat flux 25 kW/m${}^{2}$, which would lead to $T_{\mathrm{AST}}$ = 566 °C. However, such a high temperature would lead to over-prediction, originating likely from the misinterpretation between the nominal radiative source power and the real heat flux. The value $T_{\mathrm{AST}}$ = 500 °C was used further in the simulation, being much closer to the measured near-surface data.

The emissivity was assumed as 0.9, the heat transfer coefficient of the non-exposed surface as 15 W/m${}^{2}$/K. The experiment had the initial temperature of 20 °C, the timber dry bulk density of 524 kg/m${}^{3}$ and the initial moisture content of 11 wt. %. The material properties followed Figure 4. The standard properties of aluminum were considered as the bulk density of 2689 kg/m${}^{3}$, the thermal capacity of 900 J/kg/K and the thermal conductivity of 237 W/m/K. The model settings were $T_{lim}$ = 490 °C and $Q_{\mathrm{vol}}$ = 500 kW/m${}^{3}$.

**Figure 11:**
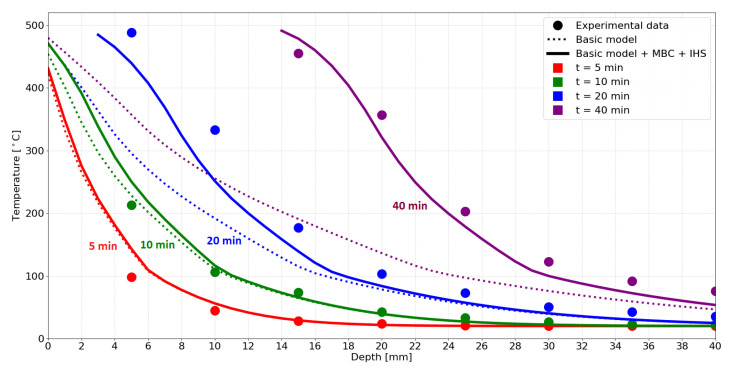
Simulation results compared with experimental data by Reszka and Torero [26].

The results depicted in Figure 11 present a very good match of the simulation and experimental data, which confirm the relevancy of MBC and IHS up to 40 min of the experiment. Focusing on the charring rate, β, the model yields almost a constant value when identifying the 300 °C temperature front. The constant value comes from the MBC formulation since the distance between $T_{lim}$ and 300 °C is rather constant.

In this simulation, the internal heat source is active for approximately 12 min, leading to a released energy of 0.5 × 12 × 60 = 360 MJ/m${}^{3}$ = 0.687 MJ/kg. This is substantially lower than the combustible energy of 20 MJ/kg, showing that only 3.5% is transferred back within the timber.

### 3.3. 2D Simulation

**Figure 12:**
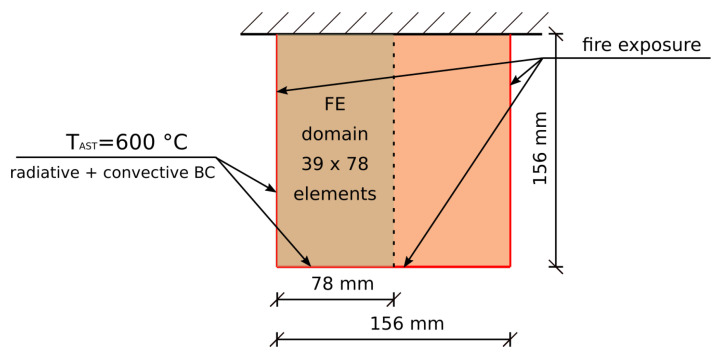
Geometry of the 2D task.

A 2D timber beam serves as an example of the corner effect. The beam’s cross section of 156×156 mm contains three surfaces exposed to fire, see the geometry in Figure 12.

The simulation assumed the constant radiative source corresponding to $T_{\mathrm{AST}}$ = 600 °C, the timber dry bulk density 500 kg/m${}^{3}$, the initial moisture content 0%, the emissivity 0.9, the heat transfer coefficient 15 W/m/K, the internal heat source $Q_{\mathrm{vol}}$ = 100 kW/m${}^{3}$ and the limit temperature $T_{lim}$ = 593 °C.

**Figure 13:**
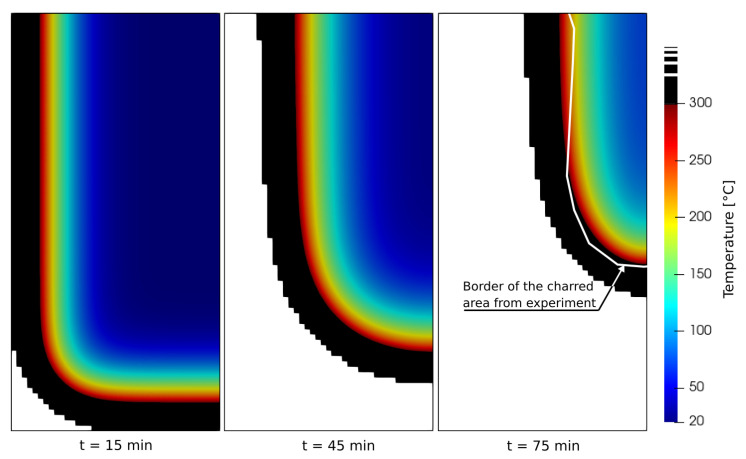
Beam exposure in 2D domain with temperature profiles. Black area = charred layer, white area = removed elements.

Figure 13 presents the results at 15, 45 and 75 min. Taking 300 °C isotherm for the onset of charring, a black area corresponds to an effective charred layer which protects the beam from direct external exposure. Similar results for 75 min were obtained by Ek [4] who used ISO 834 temperature curve instead, see white line in Figure 13.

Both the simulation and the experiment demonstrate the corner effect; charring rate proceeds slightly faster in the vertical direction since it is accelerated from the horizontal advancing front of the narrower beam. Other factors might play a role in the experiment, such as air flow, air temperature, cracking of charred layer etc.

## 4. Conclusions

The paper presents a heat transport model, complemented with a moving boundary condition and an internal heat source. The criteria for the finite element removal and boundary condition shift were successfully calibrated on several experiments. The comparison with experimental data further revealed that the moving boundary condition performed well in temperature profiles, especially if fire exposures exceeded approximately 10 min. The concept of a moving boundary condition can find its application in several other models, generally extending their validity to longer exposures.

The model yields an almost constant charring rate on fire exposures with a constant radiative power, justifying the simpler approaches used in common design methods. Extension to a 2D beam exposure captured well the evolution of the charred layer, undamaged timber core and the corner effect.

## Data Availability

All data were provided in the article.
